# Tenosynovial giant cell tumor: case report of a patient effectively treated with pexidartinib (PLX3397) and review of the literature

**DOI:** 10.1186/s13569-018-0101-2

**Published:** 2018-07-10

**Authors:** Nicholas Giustini, Nicholas M. Bernthal, Susan V. Bukata, Arun S. Singh

**Affiliations:** 1 Division of Hematology and Oncology, University of California Los Angeles (UCLA), 2825 Santa Monica Blvd. Suite 200, Santa Monica, CA 90404 USA; 2 Division of Orthopedic Oncology, University of California Los Angeles (UCLA), 1250 16th Street, Suite 2100, Santa Monica, CA 90404 USA

**Keywords:** Tenosynovial giant cell tumor, TGCT, Giant cell tumor of tendon sheath, GCT-TS, Pigmented villonodular synovitis, PVNS, Pexidartinib, PLX3397, Case report, CSF1

## Abstract

**Background:**

Tenosynovial giant cell tumors (TGCTs) or giant cell tumors of tendon sheath are neoplasms that arise in the synovium. They can be categorized as nodular (localized) or diffuse type (D-TGCT). Historically, surgery has been the mainstay of therapy, but diffuse type disease recurs at a high rate and treatment often requires increasingly morbid procedures. Elucidation of the importance of the colony-stimulating factor (CSF1)/CSF1 receptor (CSF1R) pathway in the pathogenesis of this disease has created significant interest in targeting this pathway as a novel TGCT treatment approach. Pexidartinib, a selective tyrosine kinase inhibitor against CSF1R, showed an 83% disease control rate (52% with partial response and 31% with stable disease) in a recent phase 1 study of patients with TGCT.

**Case presentation:**

We present an illustrative example of a TGCT patient who would have required a morbid operation who derived considerable clinical benefit from pexidartinib treatment. Her tumor volume decreased by 48% after 4 months of treatment, and 55 months after starting treatment the patient exhibits continued disease stability with minimal clinical symptoms, and significant improvement in functional status.

**Conclusions:**

This case illustrates the effectiveness of systemic therapy in controlling a disease associated with high surgical morbidity. This approach may be especially useful in the treatment of extra-articular disease which often invades neurovascular bundles; as the effectiveness in metastatic disease is still unknown. In the future, systemic treatment for TGCT may be appropriate for the neoadjuvant setting to decrease disease burden prior to surgery with the aim of decreasing recurrence rates. However, properly designed prospective studies will need to be carried out to answer these questions.

## Background

Tenosynovial giant cell tumor (TGCT), is a neoplasm derived from the synovium that causes recruitment of immune cells, specifically macrophages, leading to formation of a mass. These tumors are often classified by their growth pattern (localized or diffuse) and site (intra- or extra-articular). These pathological distinctions are important because variability in the clinical and biological features of these neoplasms affect treatment [1].

Localized TGCT is characterized by a discrete nodule, with a predilection for the radial three digits [2]. Diffuse TGCT (D-TGCT), formerly known as pigmented villonodular synovitis (PVNS), is characterized by a diffuse proliferation in the synovium most commonly occurring in and around the knee (~ 75% of tumors) [3]. While local and diffuse disease occur intra-articularly throughout the body, D-TGCT can also be extra-articular, and, in rare circumstances, can metastasize to adjacent lymph nodes and the lungs.

Both localized and diffuse TGCT occur primarily in the 3rd–4th decades of life with a female:male predominance of 1.6–2.1:1 in the localized type and no sex predilection in the diffuse type. The annual incidence in the United States is approximately 11 per million; of that, 9.2 being localized and 1.8 diffuse types. In a more recent epidemiological study from the Netherlands, the worldwide incidence rate of TGCT (including both localized and diffuse) was estimated to be 43 per million [4].

Clinical presentation of TGCT is variable. The first sign for patients with pathology in the hand is often a painless, slow-growing, firm nontender mass, which may eventually become painful and edematous as it impinges on anatomic structures [2]. When presenting in the knee, intermittent swelling without antecedent trauma is a common symptom [5].

In 2006, the landmark article by West et al. shed light on the pathogenesis of TGCT. Chromosomes 1 and 2 undergo a translocation at 1p13, which fuses to 2q35, leading to the fusion of *CSF1* to *COL6A3* and the overproduction of CSF1. However, in collected samples, elevated CSF1 expression was found in only 2–16% of the cells comprising the tumor. This may be in keeping with the fact that only a small fraction of the TGCT mass is considered the malignant clone. As CSF1R is present on immune cells, specifically macrophages, it has been postulated that overexpression of CSF1 from the malignant component of the tumor causes a tremendous immune infiltrate, which comprises the bulk of the tumor [6, 7].

Historically, TGCT has been treated using surgery with the consideration for adjuvant radiation. Surgical options for TGCT are partial or total synovectomy using arthroscopic or open techniques. While localized disease is readily curable with arthroscopic or open surgery, diffuse disease has shown high recurrence rates with arthroscopy (40%) and open surgery (14%). Open surgery also leads to increased morbidity in the form of stiffness and pain [8].

Newer systemic treatments are being used for TGCT ranging from less selective tyrosine kinase inhibitors, such as imatinib and nilotinib, to CSF1R inhibitors such as emactuzumab and cabiralizumab (monoclonal antibody) and pexidartinib (a selective tyrosine kinase inhibitor) with encouraging results [9]. In the only published phase 2 study of a drug for TGCT, nilotinib led to a disease stabilization rate in 90% of patients at 6 months, but only 6% of patients had a RECIST 1.1 objective response [10]. In a phase 1 study of emactuzumab, partial responses were seen in 24/28 patients and 2 patients had complete responses [11]. In particular, the CSF1R inhibitors are being evaluated in patients with cases with high operative morbidity. The case presented exemplifies the situation in which CSF1R inhibitors, namely pexidartinib, can be used to effectively reduce the disease burden in a patient with D-TGCT [12].

## Case presentation

RM is a 47-year-old woman who initially presented when she was 40 years old with 3 months of right knee pain in December 2010. At that time, a radiograph of the knee showed two lesions: a 3-cm circumscribed lytic lesion of the posterolateral distal femur thought possibly to be a nonossifying fibroma, as well as a 2-cm circumscribed lytic lesion with sclerotic margins involving the proximal tibial epiphysis. An MRI 1 month later showed lesions suspicious for TGCT; an orthopedic oncologist recommended a diagnostic arthroscopy with possible conversion to open anterior and posterior synovectomy. The patient declined and underwent a trial of oral prednisone with some symptomatic relief until October 2012 when she re-presented with worsening pain, edema, and a limp. At that time, she underwent biopsy, which was read as a likely inflammatory process. One week later the patient underwent an incisional biopsy.

**Fig. 1:**
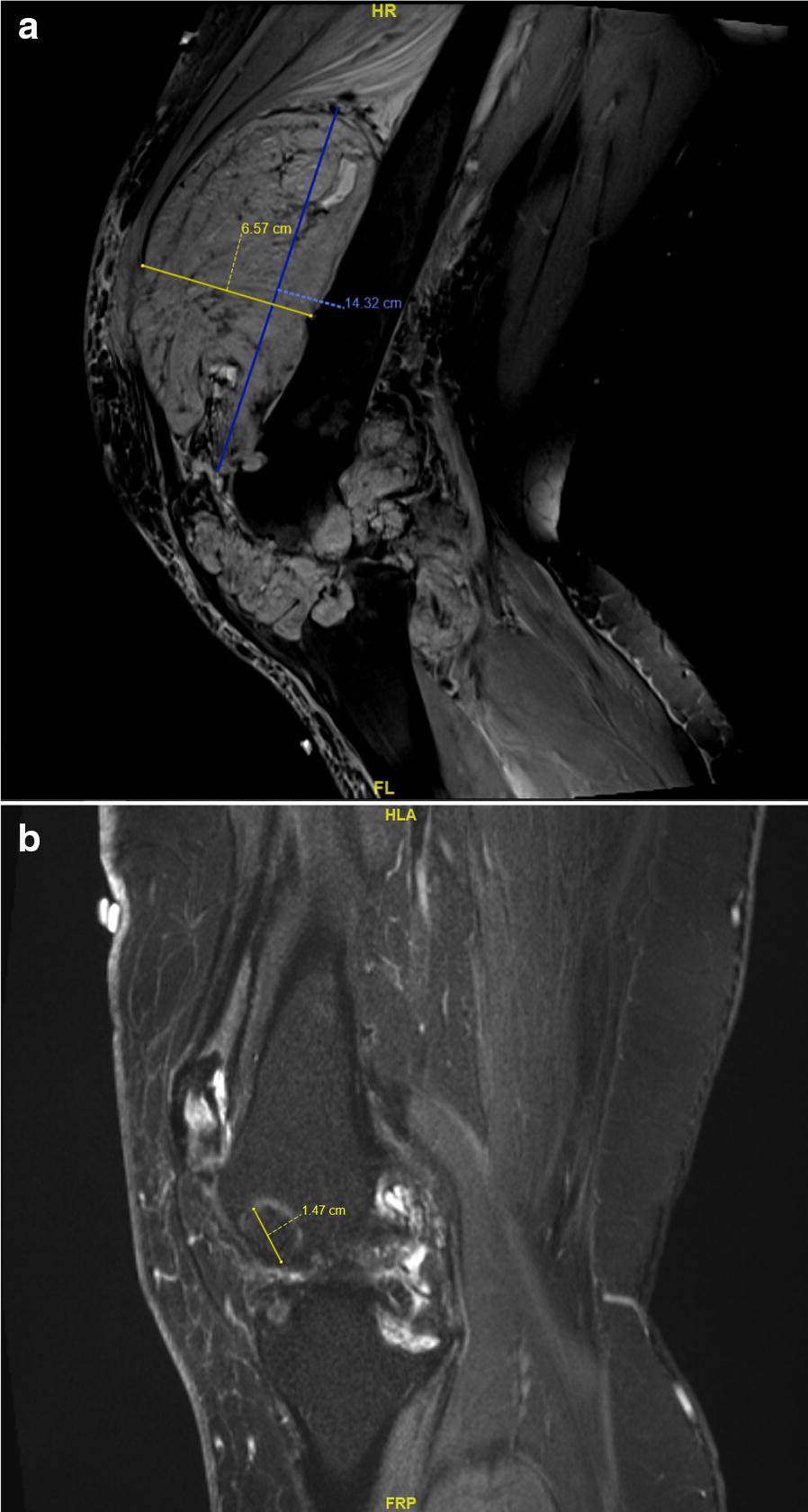
MRI scan of suprapatellar aspect in January 2013 (**a**) and May 2017 (**b**)

Histology of the neoplasm showed a hypercellular lesion consistent with D-TGCT. Immunohistochemistry revealed negative pankeratins, negative desmin, negative CD1a, positive CD68 in a patchy distribution, and a Ki-67 of 10%. A repeat MRI at the time of diagnosis showed a marked increase in the size of the synovial soft mass extending into the suprapatellar compartment, as well as anterior and posterior infrapatellar compartments. The suprapatellar aspect measured 20 cm × 9.9 cm × 13 cm (CC × AP × TV). A PET-CT scan indicated no metastatic disease and an SUV_max_ of 21.7 in the lesions (Fig. 1). Considering the extent of disease, nonsurgical management was indicated as surgical resection would likely result in significant morbidity. In February 2013, 1 week after the PET-CT, the patient consented to oral therapy with pexidartinib (PLX3397) at 600 mg qAM and 400 mg qHS for a cumulative 1000-mg daily dose. At this time, the patient had an ECOG of 1, was on disability and could no longer work as a nurse given her requirement for a walker, and was managing her pain with naproxen, morphine, and acetaminophen–hydrocodone.

At the 2-week follow-up, the patient reported an improvement of pain and an increased range of motion. Three weeks after initiation, a repeat PET-CT showed disease response with an SUV_max_ of 6.3. By 1 month after initiation, the patient reported she was transitioning to crutches and using fewer opioids. By the 4-month MRI, there was a reduction in her tumor burden by 48% via RECIST 1.1 (14.3 × 7.0 × 12.6 cm to 8.1 × 1.7 × 6.0 cm, 5.6 × 2.9 × 8.3 cm to 1.8 × 1.5 × 3.5 cm, and 9.0 × 2.4 × 9.1 cm to 5.0 × 0.8 × 3.5 cm CC × AP × TV). At this time, her ECOG was changed to 0 as she was ambulating without assistance and returned to work; she had also reduced her pain medications to occasional naproxen and had weaned herself off opioids. During the course of treatment, the dose was adjusted to 400 mg qAM and qHS for 2 months and then to 400 mg qAM and 600 mg qHS, in an effort to manage nausea. Other side effects included fatigue, dysgeusia, and peri-orbital edema. After 55 months of therapy, the patient had stable disease following the initial response with no progression at any time during the treatment course. The most recent MRI imaging on 9/8/2017 showed only two aspects of the lesion, measuring 0.4 × 4.9 cm (AP × TV with inability to visualize CC view) and 1.2 × 1.5 × 2.9 cm (CC × AP × TV).

## Discussion

In 1941, Jaffe et al. [13] initially created the category of pigmented villonodular synovitis, bursitis, and tenosynovitis, forming a classification for a seemingly diffuse group of entities. Much later, Rao and Vigorita [14] suggested that PVNS is a true neoplastic, rather than inflammatory, process involving fibroblastic or histiocytic mesenchymal cells. Subsequent research identified chromosome translocations present in TGCT cases which induce overexpression of CSF-1 in neoplastic synovial cells, in turn causing recruitment of macrophages and other immune cells form the bulk of the tumor mass [6]. Some TGCT cases do not appear to harbor such translocations, though notably they still show elevated levels CSF1 protein or RNA, suggesting there are alternate mechanisms of CSF1 overproduction [15].

Pexidartinib, or PLX3397, is a selective CSF1R inhibitor which targets the CSF1/CSF1R pathway implicated in TGCT pathogenesis. Pexidartinib was designed to simulate the autoinhibited state of the CSF1R by interacting with the CSF1R juxtamembrane region which is responsible for folding and inactivation of the kinase domain and prevents binding of CSF1 and ATP [16].

Based on an initial phase I dose escalation study evaluating the safety and tolerability of pexidartinib in patients with solid tumors, the maximum tolerated dose of 1000 mg/day was chosen for an extension study to evaluate efficacy in TGCT. This multicenter, single cohort extension study enrolled 23 patients with advanced TGCT. Eligibility requirements included a histologically confirmed diagnosis of TGCT that had shown progression within the previous year and was deemed surgically challenging (recurrent vs. inoperable vs. requiring extensive surgery for attempted resection). The mean age of patients was 46 years old with 18 patients having undergone previous surgery, four patients having previously been treated with either imatinib or nilotinib, and one patient with metastatic disease. Mean duration of treatment was 254 days with 61% of patients requiring dose reduction and 30% requiring a temporary drug withdrawal, most commonly for fatigue. A common laboratory abnormality was elevated aminotransferase levels in approximately 50% of patients (only 13% were grade 3 and all resolved to grade 1 with temporary drug cessation), thought secondary to CSF1 inhibition on Kupffer cell functioning [16].

In an intention-to-treat analysis of response using RECIST 1.1, a total of 83% of patients exhibited disease control with 52% showing partial response and 31% showing stable disease. Due to the complex shape of many TGCTs, conventional tumor response assessment methodologies are not ideal for discerning response in this disease. Tumor volume score, an unvalidated calculation of the tumor volume as a percentage of the total synovium, similar to a scoring system in use for rheumatoid arthritis, was used to evaluate 14 patients. Of these, 11 had a partial response and three had stable disease with a mean decrease in volume of 61%. Comparison of pre- and post-treatment biopsies obtained from one patient showed marked decreases in macrophages and general cellularity in the tissues. The patient with metastatic disease was the only patient who showed disease progression using RECIST 1.1, which occurred after 8 months of stable disease on therapy [16].

Our patient, RM, who presented with extensive local disease of the knee, was deemed to not be a candidate for surgery, with or without adjuvant radiation, because it would likely lead to significant unacceptable morbidity due to the extent of disease, her young age, and her profession. Given these factors, as well as the promising results in the study evaluating pexidartinib as treatment for TGCT, she was enrolled to start therapy with pexidartinib.

## Conclusions

Tenosynovial giant cell tumor is a tumor driven by CSF1 overexpression in neoplastic synovial cells. Before uncovering the pathogenesis of this disease, treatment was primarily surgical. Now systemic therapies, such as the tyrosine kinase inhibitor pexidartinib and the monoclonal antibody emactuzumab, are able to take advantage of the known molecular mechanism of TGCT pathogenesis by targeting the CSF1R. Several other CSF1R inhibitors are being evaluated clinically and a comprehensive review of this class of drugs can be found elsewhere [9]. As evidenced in our case report and in a clinical study, pexidartinib has the potential to decrease or stabilize the size of TGCT masses, reduce pain and improve mobility [16, 17].

As the use of systemic therapies such as pexidartinib become more established as treatments for TGCT, future avenues of study abound. To date, complete response with pexidartinib has not been established as the neoplastic cells are only suppressed, not removed. It has been noted that TGCT patients coming off of pexidartinib do show disease relapse (author’s unpublished observation). As such, consideration for neoadjuvant pexidartinib to allow less morbid surgical resection may be a viable option. In addition, post-surgical pexidartinib in these cases or in patients with known previous recurrence may be effective in delaying repeat recurrence. However, answers to these questions will require further validation studies of this effective targeted therapy.

## Abbreviations

TGCT: tenosynovial giant cell tumor
D-TGCT: diffuse tenosynovial giant cell tumor
CSF1: colony-stimulating factor-1
CSF1R: colony-stimulating factor-1 receptor
PVNS: pigmented villonodular synovitis
qAM: each morning
qHS: each evening
